# Genotype and Phenotype Characteristics of Chinese Pediatric Patients with Primary Hyperoxaluria

**DOI:** 10.1155/2023/4875680

**Published:** 2023-09-14

**Authors:** Yucheng Ge, Yukun Liu, Ruichao Zhan, Zhenqiang Zhao, Jun Li, Wenying Wang, Ye Tian

**Affiliations:** Department of Urology, Beijing Friendship Hospital, Capital Medical University, Beijing 100050, China

## Abstract

Primary hyperoxaluria (PH) is a rare monogenic disorder characterized by recurrent kidney stones, nephrocalcinosis, and renal impairment. To study the genotype and phenotype characteristics, we evaluated the clinical data of 42 Chinese pediatric PH patients who were diagnosed from May 2016 to April 2022. We found that patients with the PH3 type showed an earlier age of onset than those with the PH1 and PH2 types (1 versus 5 and 8 years, respectively, *P* < 0.001). Urine citrate was significantly lower in PH1 and PH2 patients than that in PH3 patients (91.81 and 85.56 versus 163.9 *μ*g/mg, respectively, *P* = 0.044). Spot urine oxalate levels were slightly higher in PH1 than that in PH2 and PH3 patients (457.9 versus 182.38 and 309.14 *μ*g/mg, respectively, *P* = 0.189). A significant negative correlation between the urine calcium/creatinine ratio and the oxalate/creatinine ratio was observed in the entire PH cohort (*r* = −0.360, *P* = 0.04) and the PH3 cohort (*r* = −0.674, *P* = 0.003). PH-causative genes showed hotspot mutations or regions, including c.815_816insGA and c.33dup in *AGXT*, 864_865del in *GRHPR*, and exon 6 skipping and c.769T>G in *HOGA1*. In the PH1 cohort, the estimated glomerular filtration rate (eGFR) was lowest in patients with heterozygous c.33dup. In the PH3 cohort, patients with heterozygous exon 6 skipping presented the lowest eGFR and a significant decrease in the renal survival advantage. In summary, PH1 patients exhibit much more severe phenotypes than those with other types. Hotspot mutations or regions exist in patients with all types of PH and show differences among ethnicities. Genotype-phenotype correlations are observed in PH1 and PH3.

## 1. Introduction

Primary hyperoxaluria (PH) is a rare autosomal recessive hereditary disease that can be divided into 3 types, namely, PH1, PH2, and PH3. PH1 is the most prevalent and severe form of hyperoxaluria, characterized by massive urinary oxalate excretion and severe renal impairment caused by a deficiency of alanine-glyoxylate aminotransferase (AGT) owing to mutations in the *AGXT* gene. To date, over 200 different pathogenic mutations have been identified in *AGXT*. The most common variant is c.508G>A, accounting for 25-40% of total alleles, which is considered associated with a relatively good prognosis of PH1 [1]. PH2 is caused by mutations in the *GRHPR* gene, which encodes the human enzyme glyoxylate reductase/hydroxypyruvate reductase (GRHPR). Renal damage progresses more slowly in PH2 patients, similar to what has been observed in PH1 patients who are responsive to pyridoxine [2], so the overall outcome of PH2 patients is generally better than that of PH1 patients. To date, approximately 40 different mutations in *GRHPR* have been reported, and the two most common variants are c.103del and c.494G>A, accounting for more than 50% of total alleles [3]. PH3 is the most recently identified type and is caused by mutations in the *HOGA1* gene, which encodes the 4-hydroxy-2-oxoglutarate aldolase (HOGA) enzyme. PH3 patients are characterized by recurrent nephrolithiasis and hypercalciuria, with earlier onset of symptoms and better renal survival. To date, over 30 different mutations have been discovered in *HOGA1*. The most prevalent variant is c.700 + 5G > T, accounting for 44% of total alleles [4].

The characteristics of PH patients in Chinese pediatric populations have not yet been comprehensively described, and very few studies on genotype-phenotype correlations are available. This study is aimed at exploring genotypic and phenotypic characteristics and possible genotype-phenotype correlations in Chinese pediatrics with PH.

## 2. Methods

This study was approved by the ethics committee of Beijing Friendship Hospital, Capital Medical University. Pediatric patients (<14 years old) who were admitted to our hospital for kidney stones were enrolled from May 2016 to April 2022. The exclusion criteria were patients who had clinical and/or laboratory evidence of potential secondary nephrolithiasis, as well as those whose parents did not consent to genetic testing. Whole-exome sequencing (WES) was performed to identify the pathogenic mutations, and the variants of the proband and his family members were validated by the Sanger sequencing. Databases such as the Human Gene Mutation Database (HGMD), Genome Aggregation Database (gnomAD), and ClinVar were used to confirm novel variants. Allele frequencies (AFs) for each mutation in various populations were obtained from gnomAD. Functional prediction of novel mutations was performed using PolyPhen-2 (http://genetics.bwh.harvard.edu/pph2/), Mutationtaster (http://mutationtaster.org), SpliceAI, and Alamut Visual Plus™. The estimated glomerular filtration rate (eGFR) was calculated by the modification of diet in renal disease (MDRD) equation. Measurements of spot urine and serum samples and renal images were performed at the time of diagnosis. We introduced the solute/creatinine ratio from spot urine as an index of urinary solute excretion. The results were expressed as medians and ranges (median (min-max)) or means ± standard deviations (means ± SDs) for continuous variables. Categorical variables were presented as percentages (%). Comparisons between groups were demonstrated with Student's *t*-test, Mann–Whitney *U* test, and a Kruskal–Wallis test for continuous variables and Pearson's chi-square test and Fisher's exact test for categorical variables. Correlations between urinary oxalate and urinary calcium were examined by the Pearson or Spearman correlation and expressed according to the Pearson or Spearman coefficient. *P* values < 0.05 were considered significant. All clinical data were analyzed using SPSS version 25.

## 3. Results

A total of 42 pediatric patients were diagnosed with PH and included in this retrospective study, including 18 with PH1 (42.86%), 7 with PH2 (16.67%), and 17 with PH3 (40.48%) (Supplementary Table S1). Among these patients, 30 (71.43%) were male (15 PH1, 3 PH2, and 12 PH3), and 12 (28.57%) were female (3 PH1, 4 PH2, and 5 PH3). The age of onset ranged from 0.3 to 13 years, and the median age was 2.75 years. The median onset age was 5 (0.3, 11) years in PH1 compared with PH2 (8 (1, 13) years) and PH3 patients (1 (0.3, 6) year) (*P* < 0.001) (Table 1). One or more spot urine tests (*n* = 63) were available in 36 patients, consisting of a single test (*n* = 14), two repeated tests (*n* = 36), three repeated tests (*n* = 9), and four repeated tests (*n* = 4).

### 3.1. Phenotypes of Chinese Pediatric Patients with PH

**Figure 1:**
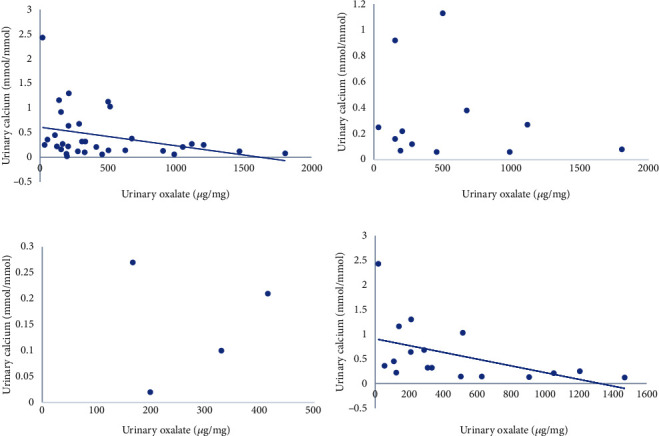
Scatterplot showing the distribution of urinary oxalate/creatinine and calcium/creatinine among all patients (a), PH1 patients (b), PH2 patients (c), and PH3 patients (d).

Nephrocalcinosis (NC) was present at diagnosis in 12 (66.67%) PH1, 3 (42.86%) PH2, and 4 (23.53%) PH3 patients (*P* = 0.105). Urine oxalate was slightly higher in PH1 than that in PH2 and PH3 patients (*P* = 0.189), while the median urine calcium was slightly lower in PH1 than that in PH2 and PH3 patients (*P* = 0.096). The median urine citrate level in PH1 (91.81 (48.79, 502.38) *μ*g/mg) and PH2 patients (85.56 (13.95, 184.78) *μ*g/mg) was significantly lower than that in PH3 patients (163.9 (46.61, 645.4) *μ*g/mg) (*P* = 0.044). Interestingly, a negative correlation between the urine calcium/creatinine ratio and the oxalate/creatinine ratio was observed in the entire PH cohort (*r* = −0.360, *P* = 0.04) and PH3 cohort (*r* = −0.674, *P* = 0.003) (Figure 1). Ultimately, 36 individuals received lithotripsies (12 PH1, 7 PH2, and 16 PH3) due to urinary tract obstruction caused by stones. However, kidney stones could not be cleared due to the massive stone burden in most PH1 and PH2 patients. Among the remaining 6 patients, three PH1 patients were treated with pyridoxine and citrate, two PH1 patients received liver transplantation, and one PH3 patient presented with small kidney stones and received close monitoring. A principal component analysis of stones revealed that calcium oxalate (CaOx) stones were present in all PH patients (*n* = 26). All PH1 (*n* = 9), PH2 (*n* = 5), and 33.3% (*n* = 4) of PH3 patients presented with calcium oxalate monohydrate (COM) stones, while 50% (*n* = 6) of PH3 patients presented with mixed COM and calcium oxalate dihydrate (COD) stones, and 16.7% (*n* = 2) of PH3 patients presented only COD stones. One month after surgery, residual stones were observed in 9 PH1 (69.23%) and 4 PH2 (57.14%) patients, but none were observed in PH3 patients (*P* < 0.001). After a mean follow-up of 4.15 years (6 months to 7 years), all PH1 and PH2 patients showed stone recurrence, while stone recurrence only occurred in 4 PH3 patients (25%) (Table 1). Fourteen patients (7 PH1, 2 PH2, and 5 PH3) had progressed to chronic kidney disease (CKD) at the time of diagnosis, of whom 10 were classified as CKD2, 3 as CKD3a, and 1 as CKD3b. During the follow-up period, further renal impairment was observed in 9 individuals, including 5 PH1, 1 PH2, and 3 PH3 patients. One PH1 patient eventually died of ESRD (Supplementary Table S1).

### 3.2. Genotypes of Chinese Pediatric Patients with PH

**Figure 2:**
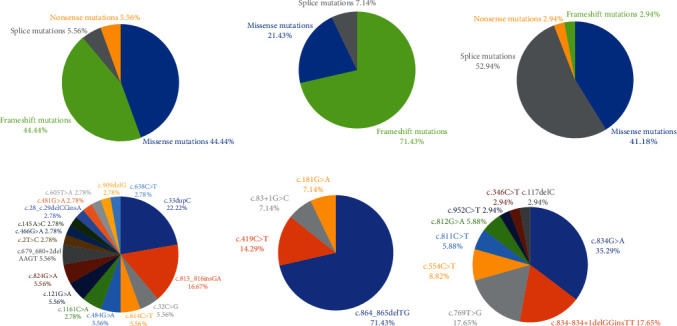
The proportion of variant types in the PH1 cohort (a), PH2 cohort (b), and PH3 cohort (c). The proportion of genotypes in the PH1 cohort (d), PH2 cohort (e), and PH3 cohort (f).

**Figure 3:**
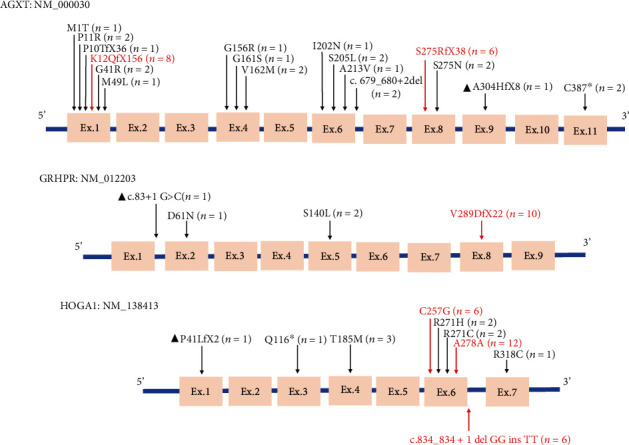
Schematic representation of known pathogenic amino acid changes associated with PH1 (a), PH2 (b), and PH3 (c) in this cohort. The “n” represents the frequency of the mutation emerged in the patients. Hotspot mutations are indicated in red, and novel mutations identified in this study are indicated with triangles.

In total, 30 different variants (17 in *AGXT*, 4 in *GRHPR*, and 9 in *HOGA1*) were identified within this cohort, 3 of which have not been reported previously (1 in *AGXT*, 1 in *GRHPR*, and 1 in *HOGA1*). In the PH1 cohort, four types of variants were found, including missense (44.44%), frameshift (44.44%), nonsense (5.56%), and splicing (5.56%) variants (Figure 2). The *AGXT* gene mutation distribution was clustered in exon 1, followed by exons 4, 6, and 8 (Figure 3). The two most common variants were c.33dup and c.815_816insGA in *AGXT*, and the AFs were 22.22% and 16.67%, respectively. The prevalence of these two mutations was highest in the East Asian (EAS) population compared to the other populations (c.33dup: EAS: 1/5110; c.815_816insGA: EAS: 3/18392). Additionally, mutations such as c.815_816insGA, c.638C>T, c.145A>C, and c.679_680+2del were also specific to the EAS population in the gnomAD database (Supplementary Table S2). All mutations except for c.909del have been previously reported, which was considered to cause premature termination of translation and was predicted to be pathogenic. In the PH2 cohort, three types of variants were found, including frameshift (71.43%), missense (21.43%), and splicing (7.14%) variations (Figure 2), and c.864_865del (AF, 71.43%) was the most common mutation in *GRHPR*, which was located in exon 8 (Figure 3). The AF of c.864_865del in the South Asian (SAS) population is 1/30614, while it is 10/19952 in the EAS population. This mutation is exclusively observed in Asian populations (Supplementary Table S2). One novel mutation was found, c.83+1G>C, which was considered likely to affect splicing and predicted to be likely pathogenic (SpliceAI predicted a donor loss of -1 bp with a score of 0.95; and Alamut predicted a change at a donor site 1 bp upstream with a score of -100.0%). In the PH3 cohort, five types of variants were found, including missense (41.18%), splicing (52.94%), nonsense (2.94%), and frameshift (2.94%) variants (Figure 2). *HOGA1* gene mutations were commonly focused in the region of exon 6 (Figure 3). c.834G>A (35.29%), c.769T>G (17.65%), and c.834_834+1delGGinsTT (17.65%) were the three commonly observed variants of *HOGA1*. The c.834G>A and c.834_834+1delGGinsTT mutations cause partial and complete exon 6 skipping, respectively, with a cumulative AF of 52.94%. The AF of these two mutations is higher in the EAS population than in the SAS population (c.834G>A: EAS: 17/19484, SAS: 2/29730; c.834_834+1delGGinsTT: EAS: 13/17924, SAS: 3/29716) (Supplementary Table S2). The c.117delC in *HOGA1* has not been reported previously and was considered to result in a frameshift and premature termination of translation. It was predicted to be pathogenic.

### 3.3. Genotype-Phenotype Correlations in PH1, PH2, and PH3 Patients

**Figure 4:**
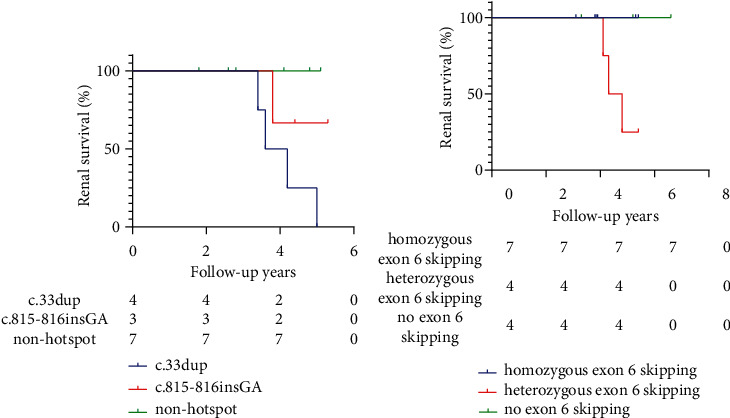
The Kaplan–Meier renal survival curves after different follow-up times (years) and among different genotypes of PH1 patients (a) and PH3 patients (b), where variants were classified as hotspot and nonhotspot variants. Tables under the Kaplan–Meier curves show the numbers of patients at potential risk corresponding to the years of follow-up.

Genotype-phenotype correlations were analyzed according to whether the patients harbored hotspot mutations or regions. In the PH1 cohort, the patients were divided into three groups: a c.33dup group, a c.815_816insGA group, and a nonhotspot mutation group. The rates of NC were 83.33%, 100%, and 42.86% in patients with c.33dup, c.815_816insGA, and nonhotspot mutations, respectively (*P* = 0.027). The lowest eGFR was observed in patients with c.33dup (69.48 ± 23.66 ml/min/1.73 m^2^), followed by those with c.815_816insGA (89.28 ± 27.31 ml/min/1.73 m^2^), and those with nonhotspot mutations (136.78 ± 23.65 ml/min/1.73 m^2^) (*P* = 0.001) (Table 2). A boy carrying a compound heterozygous mutation of c.33dup and c.679_680+2del eventually died of ESRD. When eGFR decline (<60 mL/min/1.73 m^2^) was defined as the endpoint event, the survival curves for PH1 patients were statistically significant. The patients with nonhotspot mutations had an obvious renal survival advantage (*P* = 0.0416) (Figure 4(a)). In addition, the PH1 cohort was grouped according to the presence of homozygous hotspot mutations (c.33dup homozygotes and c.815_816insGA homozygotes), heterozygous hotspot mutations (c.33dup heterozygotes and c.815_816insGA heterozygotes), and nonhotspot mutations. eGFR was lowest in c.33dup heterozygotes (63.00 ± 27.60 ml/min/1.73 m^2^) and highest in the nonhotspot mutation group (136.78 ± 23.65 ml/min/1.73 m^2^) (*P* = 0.014) (Table 3). The PH1 patients were also divided into two groups based on the mutation types, splicing/nonsense/frameshift mutations and missense mutations. The results indicated that those carrying splicing/nonsense/frameshift mutations exhibited significantly lower eGFR (79.76 ± 25.67 ml/min/1.73 m^2^ vs. 124.18 ± 32.56 ml/min/1.73 m^2^, *P* = 0.003) and a higher rate of NC (91.67% vs. 40%, *P* = 0.02) than those with missense mutations, while no significant differences were observed in other phenotypes (Supplementary Table S3).

In the PH2 cohort, only one patient did not have the c.864_865del mutation, so genotype-phenotype correlation analysis could not be performed between hotspot and nonhotspot mutations. No significant differences were found in terms of the age of onset, eGFR, urine oxalate, citrate, or calcium levels between the patients with splicing/nonsense/frameshift mutations and those with missense mutations (Supplementary Table S4).

In the PH3 cohort, the patients were divided into a homozygous exon 6 skipping group (c.834G>A/c.834G>A, c.834_834+1delGGinsTT/c.834_834+1delGGinsTT, or c.834G>A/c.834_834+1delGGinsTT), a heterozygous exon 6 skipping group (c.834G>A or c.834_834+1delGGinsTT combined with another mutation), and a no exon 6 skipping group. The eGFR was lowest in patients with heterozygous exon 6 skipping compared with patients in other groups. There was no significant difference in nephrocalcinosis, urine oxalate, citrate, or calcium levels among the three groups (Table 4). The patients with homozygous exon 6 skipping and no exon 6 skipping showed a significant renal survival advantage (Figure 4(b)), while 75% of patients with heterozygous exon 6 skipping showed risk events (*P* = 0.0437). There were no significant differences in terms of the age of onset, eGFR, urine oxalate, citrate, or calcium levels between the patients with splicing/nonsense/frameshift mutations and those with missense mutations (Supplementary Table S5).

## 4. Discussion

PH is a rare disorder; to date, 454 PH patients have been entered in the Rare Kidney Stone Consortium Primary Hyperoxaluria registry (RKSC PH registry), among which PH1, PH2, and PH3 patients account for 72%, 10%, and 9%, respectively. Regrettably, limited information about Chinese patients with PH has been reported [5, 6]. To our knowledge, we have assembled the largest cohort of Chinese pediatric patients with PH reported to date, analyzed the data of 42 subjects, demonstrated the genotypes and clinical course, and investigated possible genotype-phenotype correlations.

### 4.1. Phenotypic Characteristics of PH

We noted that there were similar numbers of PH1 and PH3 patients in our cohort, which indicated that PH3 is not as uncommon in China as previously thought. The PH3 variant carrier frequency is estimated to be 1 : 185 [7], suggesting that there are a large number of potential PH3 patients and that mass screening will be needed to identify morbidity caused by this type. A trend toward earlier first symptoms was observed for PH3 compared with PH1 and PH2 [8]. Consistent with previous studies, the earliest clinical signs were seen in PH3 patients in the present study. Singh et al. found that severe renal phenotypes were more commonly identifiable in PH1 patients. NC was observed in 26-76.92% of PH1 patients compared with 16-35% of PH2 and 7% of PH3 patients, which was considered an independent risk factor for kidney damage and more likely to deteriorate in PH1 [2, 8–11]. In our study, NC was also highest in PH1 patients (66.67%), followed by PH2 patients (42.86%), and the NC rate in PH3 patients was not as low (23.53%) as expected. Severe renal phenotypes are usually rare in PH3 patients, and there may be a shift toward an improvement in PH3 patients with increasing age [12]. A previous study indicated that PH1 patients presented the lowest eGFR and that PH3 patients presented the highest [8]. However, this trend was not observed in our cohort, possibly because of the relatively small sample size.

Regarding urinary chemistry, Martin-Higueras et al. [13] found that urinary oxalate excretion of PH3 patients (1.37 mmol/1.73m^2^/24 h) was not significantly different from PH1 patients not responsive to vitamin B6 (1.40 mmol/1.73m^2^/24 h) and PH2 patients (1.16 mmol/1.73m^2^/24 h) but was significantly higher than PH1 patients responsive to vitamin B6 (0.94 mmol/1.73m^2^/24 h). In contrast, Singh et al. [8] reported that urine oxalate excretion (1.6 mmol/1.73 m^2^/24 h) was significantly higher in PH1 than in PH2 (1.5 mmol/1.73 m^2^/24 h) and PH3 patients (1.1 mmol/1.73 m^2^/24 h), while urine calcium and urine citrate excretion were lowest in PH1 patients. In the present study, we also observed that PH1 patients presented the highest urine oxalate, lowest urine calcium, and lowest citrate levels, while the opposite was true in PH3 patients. Moreover, hyperoxaluria was more likely to promote COM formation, and hypercalciuria was more likely to promote COD formation [14]. The significant negative correlation between urine calcium and urine oxalate observed in the entire PH cohort and the PH3 cohort in our study could further explain the different stone compositions among the three types of PH. The higher ratio of urine calcium to oxalate found in PH3 (0.001, 0.32/309.14) versus PH1 patients (0.0004, 0.19/457.9) may favor a higher rate of COD (mixed COM and COD in 50% of PH3, COD in 16.7% of PH3 versus no COD in PH1). Increased urinary oxalate excretion is considered the leading cause of urolithiasis, NC, and renal impairment, which could be used as a prognostic predictor in patients with PH [15–17]. Waikar et al. [18] found that patients with much higher urinary oxalate excretion showed a 33% higher risk of CKD progression and a 45% higher risk of ESRD than those with lower oxalate excretion. Up to 64% of PH1 patients show progression to ESRD by 40 years of age, compared to 34% of PH2 and 3% of PH3 patients [8]. PH3 patients rarely exhibit severe renal impairment, and only three PH3 patients have progressed to ESRD to date [7, 19]. In this study, renal impairment was observed in 27.77% of PH1, 14.29% of PH2, and 17.65% of PH3 patients during the follow-up. One patient progressed to ESRD in PH1, and no ESRD occurred in PH2 or PH3 patients.

### 4.2. Genotypic Characteristics of PH Patients

For PH1 patients, missense mutations were the most common type identified in the Caucasian population. Some frequent mutations, such as c.508G>A, c.454T>A, c.121G>C, and c.731T>C, typically lead to mistargeting of the AGT enzyme to the mitochondria and result in a 30% reduction in AGT activity [20]. In the present study cohort, both missense and frameshift mutations were common types in AGXT. The three most prevalent variants, c.508G>A, c.33dup, and c.731T>C, are commonly seen in European and North American populations, while c.731T>C and c.33dup are the most common variants in Tunisia and Syria, respectively [7, 21–23]. To date, three variants have been identified as possible hotspot mutations in the Chinese population, namely, c.33dup, c.815_816insGA, and c.679_680delAA, and c.815_816insGA is the main variant identified in the Chinese population [6, 11]. In this cohort, c.33dup and c.815_816insGA are also the two most common variants. Moreover, our study cohort included two patients harboring the c.679_680+2del (AF = 5.56%) variant, which is considered exclusive to the Chinese population [24, 25].

For PH2 patients, deletion is the most common mutation type in Chinese and Caucasian populations. The common variants of *GRHPR* include c.103delG, c.494G>A, c.403_404+2delAAGT, and c.864_865del. Among these variants, c.103delG is the most common, accounting for 37.8% of the total, and it has been identified only in Caucasians. The c.494G>A and c.403_404+2delAAGT variants accounted for 15% and 10% of the total, respectively, and were mainly from the Indian subcontinent. c.864_865del is a hotspot mutation in Chinese and Japanese populations derived from a common founder variant, with an AF of 75% [3]. Four different mutations of *GRHPR* were identified in the present study, and c.864_865del accounted for 71.43% of the total alleles. Furthermore, in our study, only one patient did not carry this variant. Thus, it is believed that c.864_865del is the hotspot mutation of PH2 patients in China.

The common variant types of the *HOGA1* gene identified in our center (missense and splicing) markedly differ from those reported in Western countries (deletion and splicing). The two most prevalent mutations, namely, c.700+5G>T and c.944_946delAGG, account for 70% of total alleles worldwide. c.700+5G>T is most prevalent in Caucasians (AF, 63%) and is rarely reported in Chinese populations. The c.834_834+1 mutation region, including c.834G>A and c.834_834+1delGGinsTT mutations, has been identified to cause partial or complete exon 6 skipping. It has been previously shown that exon 6 skipping represented a potential hotspot mutation in the Chinese population, with a cumulative AF of 62% [4, 26, 27]. Similarly, the cumulative AF of exon 6 skipping in the *HOGA1* sequence in our study cohort was 52.94% (including 35.29% for c.834G>A and 17.65% for c.834_834+1delGGinsTT). These findings further emphasize the significance of the c.834_834+1 region as a crucial functional domain of the HOGA protein [28]. It is still noteworthy that a high frequency of the c.769T>G (17.65%) variant was found in our PH3 cohort, predominantly within the EAS population. Therefore, we inferred that it might be a latent hotspot mutation for Chinese PH3 patients.

### 4.3. Genotype-Phenotype Correlations in PH1, PH2, and PH3

Harambat et al. [1] found that ESRD was delayed in PH1 patients with c.508G>A and that ESRD occurred in homozygotes later than in heterozygotes. Patients with c.33dup inevitably develop severe symptoms and show early death in childhood, even when it occurs in heterozygosity [29]. Studies indicated that all pediatric patients with c.33dup progressed to ESRD at a median age of 3 years, and 75% of these individuals ultimately died of their disease at a median age of 2.5 years [30]. PH1 patients with c.33dup and c.815_816insGA in the Chinese population showed a worse prognosis [6]. Similarly, this study showed that c.33dup can cause devastating consequences, reflected in the lowest eGFR, highest urine oxalate excretion, and lowest urine calcium levels. Moreover, one pediatric patient in our cohort carrying a compound heterozygous mutation of c.33dup and c.679_680+2del presented significant deterioration of renal function with one year of follow-up. The patient eventually progressed to ESRD and died at age of 9 years. c.815_816insGA is also expected to result in the absence or disruption of the protein product and to cause severe damage in patients. One patient with c.815_816insGA, presenting at age of 3, progressed rapidly to ESRD at age of 10.6 [31]. In our study cohort, patients with c.815_816insGA presented with different degrees of renal impairment but did not progress to ESRD or early death.

Mandrile et al. [32] classified *AGXT* gene variants into nonsense, missense leading to mistargeting (p.G170R), and other missense variants. Those patients with the p.G170R/nonsense variant showed the earliest age of ESRD development, followed by those with the p.G170R/missense variants and p.G170R homozygotes. In a study by Hopp et al., patients with two minor allele-requiring variants showed milder phenotypes than those with any other genotypes, and p.G170R homozygotes also presented a renal survival advantage [7]. In our study, the patients with c.33dup had the lowest eGFR and were most likely to develop ESRD. Patients carrying hotspot mutations, c.815_816insGA or c.33dup, showed a more severe phenotype in compound heterozygotes than homozygotes. Survival curves showed a significant separation trend in the renal survival advantage among the three groups. The patients with nonhotspot mutations showed the greatest survival advantage, followed by the patients with c.815_816insGA and c.33dup. It is also worth mentioning that those carrying splicing/nonsense/frameshift mutations exhibit significantly lower eGFR and a higher rate of NC than those with missense mutations, which suggests that more severe phenotypes of PH1 patients may be associated with mutations causing the absence of protein.

Genotype-phenotype associations of PH2 patients have rarely been reported worldwide. Phenotypes do not always differ among individuals with the three major genotypes of *GRHPR* (c.103del, c.494G>A, and c.403_404+2delAAGT) [2]. To date, studies on the clinical characteristics of patients with c.864_865del have been limited, and the associated phenotypes have varied widely. One patient presented with severe renal impairment and eventually progressed to ESRD [33], and another maintained normal renal function at age of 19 years despite showing elevated serum and urine oxalate levels [34]. In the present study, renal impairment occurred in two patients with c.864_865del mutations. Regrettably, it was difficult to explore the genotype-phenotype associations of PH2 patients in our study since the sites of *GRHPR* variants were very concentrated.

To date, most genotypes identified in PH3 patients have not been associated with severe phenotypes. Sikora et al. [35] found that all patients with c.700+5G>T retained normal renal function, and only one-third of these patients showed recurrent urolithiasis. Other common mutations (c.860G>T, c.944_946delAGG, and c.907C>T) in *HOGA1* are also considered associated with milder phenotypes [36, 37]. However, we noted that hotspot mutations (exon 6 skipping) seemed to be associated with relatively severe phenotypes. In the present study, higher NC, a lower eGFR, lower calcium excretion, higher urinary oxalate excretion, and higher stone recurrence were found in patients with exon 6 skipping, and patients with heterozygous exon 6 skipping may show more severe phenotypes. It has been proposed that CKD or ESRD in PH3 patients is not necessarily associated with hyperoxaluria [15, 28, 38]. However, in this study, we found that renal function may not be as unaffected in PH3 patients as previously reported in Chinese patients [26]. Survival curves showed that the renal survival advantage of the heterozygous exon 6 skipping group was significantly lower than that of the other two groups. This suggests that exon 6 skipping, which is a hotspot mutation in Chinese PH3 patients, may be associated with more severe phenotypes and a worse prognosis.

Our study has some limitations. The first possible limitation is that the accuracy of urinary solute excretion measurement using spot urine samples remains contested. However, it is difficult to collect 24 h urine samples from pediatric patients, and it has been suggested that the solute-to-creatinine ratio obtained from spot urine analysis is a better index of solute excretion since it is simple to determine and avoids unexpected distortion caused by inaccurately timed collection, especially in children [39, 40]. The second possible issue is that the relatively short duration of follow-up in our study limits our conclusions, especially regarding long-term kidney function. The third limitation is that our relatively small sample size and some missing data lead to a lack of statistical significance. Thus, further studies with large samples and long-term follow-up are needed to provide a full characterization of PH patients in China.

## 5. Conclusion

Among Chinese pediatric patients with PH, PH1 was the most common type identified, followed by PH3 and PH2. PH3 patients showed the earliest onset, and PH2 patients showed the latest onset. PH1 patients had more severe phenotypes than PH2 and PH3 patients. Hotspot mutations or regions could be found in PH-causative genes, and ethnic differences were demonstrated. Genotypes and phenotypes may be correlated in PH1 and PH3 patients, respectively, and *AGXT* c.33dup and *HOGA1* exon 6 skipping may lead to more severe phenotypes.

## Figures and Tables

**Table 1:** Comparisons of phenotypes among PH1, PH2, and PH3 patients.

Variables	PH1 (*n* = 18)	PH2 (*n* = 7)	PH3 (*n* = 17)	*P*
Age of onset (years)	5 (0.3, 11) (*n* = 18)	8 (1, 13) (*n* = 7)	1 (0.3, 6) (*n* = 17)	<0.001^∗^
Nephrocalcinosis (%)	66.67% (*n* = 18)	42.86% (*n* = 7)	23.53% (*n* = 17)	0.105
eGFR (ml/min/1.73 m^2^)	101.85 ± 38.61 (*n* = 17)	109.96 ± 27.61 (*n* = 7)	100.12 ± 29.98 (*n* = 16)	0.807
Urine oxalate/creatinine ratio (*μ*g/mg) (nL < 253.88)	457.9 (34.02, 1860.88) (*n* = 13)	182.38 (1.79, 414.24) (*n* = 6)	309.14 (18.57, 1470.16) (*n* = 17)	0.189
Urine calcium/creatinine ratio (mmol/mmol) (nL < 0.57)	0.19 (0.06, 1.13) (*n* = 12)	0.21 (0.02 0.41) (*n* = 5)	0.32 (0.12, 2.43) (*n* = 17)	0.096
Urine citrate/creatinine ratio (*μ*g/mg) (nL > = 45.62)	91.81 (48.79, 502.38) (*n* = 10)	85.56 (13.95, 184.78) (*n* = 6)	163.9 (46.61, 645.4) (*n* = 17)	0.044^∗^
Residual stones (%)	69.23% (*n* = 13)	57.14% (*n* = 7)	0% (*n* = 16)	<0.001^∗^
Stone recurrence (%)	100% (*n* = 4)	100% (*n* = 3)	25% (*n* = 16)	<0.001^∗^

Abbreviations: nL: normal limit; eGFR: estimated glomerular filtration rate. ^∗^*P* ≤ 0.05.

**Table 2:** Phenotypes of PH1 patients with hotspot and nonhotspot mutations.

Variables	c.33dup group (*n* = 6)	c.815_816insGA group (*n* = 5)	Nonhotspot mutation group (*n* = 7)	*P*
Age of onset (years)	5.5 (3, 10) (*n* = 6)	4 (0.6, 11) (*n* = 5)	4 (0.3, 9) (*n* = 7)	0.558
Nephrocalcinosis (%)	83.33% (*n* = 6)	100% (*n* = 5)	42.86% (*n* = 7)	0.027^∗^
eGFR (ml/min/1.73 m^2^)	69.48 ± 23.66 (*n* = 6)	89.28 ± 27.31 (*n* = 4)	136.78 ± 23.65 (*n* = 7)	0.001^∗^
Urine oxalate/creatinine ratio (*μ*g/mg) (nL < 253.88)	906.07 ± 118.04 (*n* = 2)	746.58 ± 707.26 (*n* = 5)	308.75 ± 237.94 (*n* = 6)	0.228
Urine calcium/creatinine ratio (mmol/mmol) (nL < 0.57)	0.06 (*n* = 1)	0.08 (0.06, 0.27) (*n* = 5)	0.32 (0.12, 1.31) (*n* = 6)	0.058
Urine citrate/creatinine ratio (*μ*g/mg) (nL > = 45.62)	113.33 (70.33, 156.32) (*n* = 2)	91.81 (48.79, 502.38) (*n* = 4)	95.71 (66.1, 302.32) (*n* = 4)	0.960

Abbreviations: nL: normal limit; eGFR: estimated glomerular filtration rate. ^∗^*P* ≤ 0.05.

**Table 3:** Phenotypes of PH1 patients with hotspot homozygous mutations, hotspot heterozygous mutations, and nonhotspot mutations.

Variables	c.33dup homozygote	c.33dup heterozygote	c.815_816insGA homozygote	c.815_816insGA heterozygote	Nonhotspot mutations	*P*
	(*n* = 2)	(*n* = 4)	(*n* = 1)	(*n* = 4)	(*n* = 7)	
Age of onset (years)	7 (6, 8)	5 (3, 10)	1	5 (0.6, 11)	4 (0.3, 9)	0.622
	(*n* = 2)	(*n* = 4)	(*n* = 1)	(*n* = 4)	(*n* = 7)	
Nephrocalcinosis (%)	100%	75.00%	100%	100%	28.57%	0.678
	(*n* = 2)	(*n* = 4)	(*n* = 1)	(*n* = 4)	(*n* = 7)	
eGFR (ml/min/1.73 m^2^)	82.44 ± 3.08	63.00 ± 27.60	116.33	80.26 ± 25.12	136.78 ± 23.65	0.014^∗^
	(*n* = 2)	(*n* = 4)	(*n* = 1)	(*n* = 3)	(*n* = 7)	
Urine oxalate/creatinine (*μ*g/mg) (nL < 253.88)	ND	906.07 ± 118.04	1118.69	653.55 ± 780.56	308.75 ± 237.94	0.203
	(*n* = 0)	(*n* = 2)	(*n* = 1)	(*n* = 4)	(*n* = 6)	
Urine calcium/creatinine (mmol/mmol) (nL < 0.57)	ND	0.06	0.27	0.08 (0.06, 0.16)	0.32 (0.12, 1.13)	0.058
	(*n* = 0)	(*n* = 1)	(*n* = 1)	(*n* = 4)	(*n* = 6)	
Urine citrate/creatinine (*μ*g/mg) (nL > = 45.62)	70.33	156.32	502.38	83.63 (48.79, 99.98)	95.71 (66.10, 302.32)	0.420
	(*n* = 1)	(*n* = 1)	(*n* = 1)	(*n* = 3)	(*n* = 4)	

Abbreviations: nL: normal limit; eGFR: estimated glomerular filtration rate; ND: not detected. ^∗^*P* ≤ 0.05.

**Table 4:** Phenotypes of PH3 patients with and without exon 6 skipping.

Variables	Homozygous exon 6 skipping	Heterozygous exon 6 skipping	No exon 6 skipping variants	*P*
	(*n* = 7)	(*n* = 4)	(*n* = 6)	
Age of onset (years)	1.5 (0.4, 6)	0.7 (0.6, 0.8)	1.25 (0.4, 5.0)	0.494
	(*n* = 7)	(*n* = 4)	(*n* = 6)	
Nephrocalcinosis (%)	14.29%	50.00%	16.67%	0.479
	(*n* = 7)	(*n* = 4)	(*n* = 6)	
eGFR (ml/min/1.73 m^2^)	99.94 (93.50, 167.02)	70.11 (60.84, 79.66)	102.48 (55.95, 130.96)	0.050^∗^
	(*n* = 7)	(*n* = 4)	(*n* = 5)	
Urine oxalate/creatinine (*μ*g/mg) (nL < 253.88)	515.88 (110.08, 1205.59)	272.77(124.27, 906.77)	249.13 (18.57, 1470.16)	0.577
	(*n* = 7)	(*n* = 4)	(*n* = 6)	
Urine calcium/creatinine (mmol/mmol) (nL < 1.5)	0.32 (0.14, 1.16)	0.27 (0.13, 1.30)	0.50 (0.12, 2.43)	0.920
	(*n* = 7)	(*n* = 4)	(*n* = 6)	
Urine citrate/creatinine (*μ*g/mg) (nL > = 45.62)	297.93(140.61, 518.25)	136.07 (46.61, 252.97)	148.76 (89.76, 654.4)	0.096
	(*n* = 7)	(*n* = 4)	(*n* = 6)	
Stone recurrence (%)	28.57%	25.00%	16.67%	1.000
	(*n* = 7)	(*n* = 4)	(*n* = 6)	

Abbreviations: nL: normal limit; eGFR: estimated glomerular filtration rate. ^∗^*P* ≤ 0.05.

## Data Availability

The data that supports the findings of this study are available in this article.

## Supplementary Materials

Supplementary Materials Supplementary Table S1: PH patient demographics, genotypes, phenotypes, and treatment methods. Supplementary Table S2: allele frequency of PH patient mutations in this cohort and the gnomAD database. Supplementary Table S3: phenotypes of PH1 patients with splicing/nonsense/frameshift mutations and missense mutations. Supplementary Table S4: phenotypes of PH2 patients with splicing/nonsense/frameshift mutations and missense mutations. Supplementary Table S5: phenotypes of PH3 patients with splicing/nonsense/frameshift mutations and missense mutations.
