# Supplementary material for: Genotype and Phenotype Characteristics of Chinese Pediatric Patients with Primary Hyperoxaluria
Source: Hum Mutat. 2023 Sep 14;2023:4875680. doi: 10.1155/2023/4875680 (PMC11918535; doi:10.1155/2023/4875680)
Supplement: Supplementary Materials — Supplementary Table S1: PH patient demographics, genotypes, phenotypes, and treatment methods. Supplementary Table S2: allele frequency of PH patient mutations in this cohort and the gnomAD database. Supplementary Table S3: phenotypes of PH1 patients with splicing/nonsense/frameshift mutations and missense mutations. Supplementary Table S4: phenotypes of PH2 patients with splicing/nonsense/frameshift mutations and missense mutations. Supplementary Table S5: phenotypes of PH3 patients with splicing/nonsense/frameshift mutations and missense mutations. [file 4875680.f1.docx]

| Supplementary Table S1. PH patient demographics, genotypes, phenotypes and treatment methods | | | | | | | | | | | | |
| --- | --- | --- | --- | --- | --- | --- | --- | --- | --- | --- | --- | --- |
| Patients | Gene | Genotype | Position | Age of onset | Sex | eGFR at diagnosis | eGFR at the end of follow-up | Uox/Ucr | Uca/Ucr | Urine citrate/Ucr | Treatment | Follow-up |
|  |  |  |  |  |  | (ml/min/1.73m^2^) | (ml/min/1.73m^2^) | (μg/mg) | (mmol/mmol) | (μg/mg) |  | years |
| P1 | AGXT | [c.33dup (p. K12QfX156)] | exon1 | 5 | male | 34.45 | ND | ND | ND | ND | RIRS | 1.0 |
|  |  | [c.679_680+2del (splice)] | intron 6 |  |  |  |  |  |  |  |  |  |
| P2 | AGXT | [c.32C>G (p.P11R)] | exon1 | 11 | male | 88.20 | 53.38 | 194.38 | 0.07 | 99.98 | Liver transplantation | 3.8 |
|  |  | [c.815_816insGA | exon8 |  |  |  |  |  |  |  |  |  |
|  |  | (p. L272RfX38)] |  |  |  |  |  |  |  |  |  |  |
| P3 | AGXT | [c.614C>T (p.S205L)] | exon6 | 7 | male | 148.47 | 126.43 | ND | ND | ND | RIRS | 4.8 |
|  |  | [c.614C>T (p.S205L)] | exon6 |  |  |  |  |  |  |  |  |  |
| P4 | AGXT | [c.33dup (p. K12QfX156)] | exon1 | 3 | male | 97.25 | 56.38 | 822.6 | ND | ND | PCNL | 4.2 |
|  |  | [c.1161C>A (p.C387*)] | exon11 |  |  |  |  |  |  |  |  |  |
| P5 | AGXT | [c.484G>A (p.V162M)] | exon4 | 1 | male | 111.78 | 104.68 | 278.27 | 0.12 | 121.72 | RIRS | 5.1 |
|  |  | [c.484G>A (p.V162M)] | exon4 |  |  |  |  |  |  |  |  |  |
| P6 | AGXT | [c.33dup (p. K12QfX156)] | exon1 | 8 | male | 80.26 | 52.24 | ND | ND | ND | RIRS | 5.0 |
|  |  | [c.33dup (p. K12QfX156)] | exon1 |  |  |  |  |  |  |  |  |  |
| P7 | AGXT | [c.2T>C (p.M1T)] | exon1 | 7 | female | 140.56 | 142.05 | 207.22 | 0.22 | ND | RIRS | 4.8 |
|  |  | [c.605T>A (p.I202N)] | exon6 |  |  |  |  |  |  |  |  |  |
| P8 | AGXT | [c.466G>A (p.G156R)] | exon4 | 4 | male | 103.29 | 139.26 | 154.51 | 0.92 | 302.32 | PCNL | 4.1 |
|  |  | [c.1161C>A (p.C387*)] | exon11 |  |  |  |  |  |  |  |  |  |
| P9 | AGXT | [c.815_816insGA | exon8 | 4 | male | 100.47 | 133.46 | 1806.88 | 0.08 | ND | RIRS | 4.4 |
|  |  | (p. L272RfX38)] | exon9 |  |  |  |  |  |  |  |  |  |
|  |  | [c.909del (p. A304HfX8)] |  |  |  |  |  |  |  |  |  |  |
| P10 | AGXT | [c.33dup (p. K12QfX156)] | exon1 | 5 | male | 72.02 | 50.99 | 989.53 | 0.06 | 156.32 | RIRS | 3.6 |
|  |  | [c.824G>A (p.S275N)] | exon8 |  |  |  |  |  |  |  |  |  |
| P11 | AGXT | [c.815_816insGA (p. L272RfX38)] | exon8 | 1 | male | 116.33 | 122.89 | 1118.69 | 0.27 | 502.38 | Drug therapy | 5.3 |
|  |  | [c.815_816insGA (p. L272RfX38)] | exon8 |  |  |  |  |  |  |  |  |  |
| P12 | AGXT | [c.33dup (p. K12QfX156)] | exon1 | 6 | male | 84.62 | 58.9 | ND | ND | 70.33 | Drug therapy | 3.4 |
|  |  | [c.33dup (p. K12QfX156)] | exon1 |  |  |  |  |  |  |  |  |  |
| P13 | AGXT | [c.28_c.29delCCinsA | exon1 | 6 | male | 52.14 | 78.33 | 457.9 | 0.06 | 83.63 | RIRS | 5.5 |
|  |  | (p. P10TfX36)] |  |  |  |  |  |  |  |  |  |  |
|  |  | [c.815_816insGA | exon8 |  |  |  |  |  |  |  |  |  |
|  |  | (p. L272RfX38)] |  |  |  |  |  |  |  |  |  |  |
| P14 | AGXT | [c.638C>T (p.A213V)] | exon6 | 2 | male | 132.13 | 110.97 | 501.93 | 1.13 | ND | Liver transplantation | 2.6 |
|  |  | [c.481G>A (p.G161S)] | exon4 |  |  |  |  |  |  |  |  |  |
| P15 | AGXT | [c.121G>A (p.G41R)] | exon1 | 9 | male | 148.31 | 160.74 | 676.53 | 0.38 | 69.7 | PCNL | 2.8 |
|  |  | [c.121G>A (p.G41R)] | exon1 |  |  |  |  |  |  |  |  |  |
| P16 | AGXT | [c.32C>G (p.P11R)] | exon1 | 0.3 | female | 172.89 | 112.84 | 34.02 | 0.25 | 66.1 | Drug therapy | 1.8 |
|  |  | [c.145A>C (p.M49L)] | exon1 |  |  |  |  |  |  |  |  |  |
| P17 | AGXT | [c.679_680+2del (splice)] | exon6/  intron  6 | 10 | female | 48.29 | 76.93 | ND | ND | ND | PCNL | 2.4 |
|  |  | [c.33dup (p. K12QfX156)] | exon1 |  |  |  |  |  |  |  |  |  |
| P18 | AGXT | [c.815_816insGA | exon8 | 0.6 | male | ND | ND | 155.03 | 0.16 | 48.79 | Drug therapy | 1.0 |
|  |  | (p. L272RfX38)] |  |  |  |  |  |  |  |  |  |  |
|  |  | [c.824G>A (p.S275N)] | exon8 |  |  |  |  |  |  |  |  |  |
| P19 | GRHPR | [c.864_865del (p.V289DfX22)] | exon8 | 8 | female | 103.89 | 98.31 | 414.24 | 0.21 | 163.83 | RIRS | 7.0 |
|  |  | [c.864_865del (p.V289DfX22)] | exon8 |  |  |  |  |  |  |  |  |  |
| P20 | GRHPR | [c.181G>A (p.D61N)] | exon2 | 9 | male | 80.95 | 120.82 | 328.9 | 0.1 | 40.89 | RIRS | 5.5 |
|  |  | [c.864_865del (p.V289DfX22)] | exon8 |  |  |  |  |  |  |  |  |  |
| P21 | GRHPR | [c.83+1G>C (splice)] | intron1 | 13 | female | 140.94 | 131.05 | 198.36 | 0.02 | 130.23 | PCNL | 3.5 |
|  |  | [c.419C>T (p.S140L)] | exon5 |  |  |  |  |  |  |  |  |  |
| P22 | GRHPR | [c.864_865del (p.V289DfX22)] | exon8 | 1 | female | 109.89 | ND | ND | 0.41 | ND | RIRS | 4.3 |
|  |  | [c.864_865del (p.V289DfX22)] | exon8 |  |  |  |  |  |  |  |  |  |
| P23 | GRHPR | [c.864_865del (p.V289DfX22)] | exon8 | 5 | male | 137.50 | 119.37 | 166.4 | 0.27 | 184.78 | PCNL | 3.0 |
|  |  | [c.864_865del (p.V289DfX22)] | exon8 |  |  |  |  |  |  |  |  |  |
| P24 | GRHPR | [c.864_865del (p.V289DfX22)] | exon8 | 9 | male | 127.48 | 111.19 | 11.34 | ND | 36.08 | RIRS | 1.4 |
|  |  | [c.864_865del (p.V289DfX22)] | exon8 |  |  |  |  |  |  |  |  |  |
| P25 | GRHPR | [c.864_865del (p.V289DfX22)] | exon8 | 7 | female | 69.10 | 52.92 | 1.79 | ND | 13.95 | RIRS | 1.6 |
|  |  | [c.419C>T (p.S140L)] | exon5 |  |  |  |  |  |  |  |  |  |
| P26 | HOGA1 | [c.834_834＋1 del GG ins TT (splice)] | exon6-intron6 | 1 | female | 120.42 | 122.3 | 139.94 | 1.16 | 323.04 | Ureteroscopy | 3.1 |
|  |  | [c.834_834＋1 del GG ins TT (splice)] | exon6-intron6 |  |  |  |  |  |  |  |  |  |
| P27 | HOGA1 | [c.769T>G (p.C257G)] | exon6 | 1 | male | 102.48 | 133.02 | 209.68 | 0.64 | 159.22 | RIRS | 6.6 |
|  |  | [c.811C>T (p.R271C)] | exon6 |  |  |  |  |  |  |  |  |  |
| P28 | HOGA1 | [c.834G>A (splice)] | exon6 | 0.6 | female | 79.66 | 134.99 | 124.27 | 0.22 | 111.21 | Ureteroscopy, RIRS | 5.4 |
|  |  | [c.952C>T (p.R318C)] | exon7 |  |  |  |  |  |  |  |  |  |
| P29 | HOGA1 | [c.769T>G (p.C257G)] | exon6 | 0.5 | male | 55.95 | 54.47 | 1470.16 | 0.12 | 654.4 | RIRS | 6.8 |
|  |  | [c.812G>A (p.R271H)] | exon6 |  |  |  |  |  |  |  |  |  |
| P30 | HOGA1 | [c.834G>A (splice)] | exon6 | 6 | male | 99.94 | 147.16 | 110.08 | 0.45 | 140.61 | Ureteroscopy, RIRS | 5.4 |
|  |  | [c.834G>A (splice)] | exon6 |  |  |  |  |  |  |  |  |  |
| P31 | HOGA1 | [c.769T>G (p.C257G)] | exon6 | 0.7 | male | 77.64 | 58.28 | 211.83 | 1.3 | 46.61 | RIRS | 4.1 |
|  |  | [c.834G>A (splice)] | exon6 |  |  |  |  |  |  |  |  |  |
| P32 | HOGA1 | [c.834G>A (splice)] | exon6 | 0.5 | male | 93.50 | 128.18 | 515.88 | 1.03 | 349.06 | RIRS | 5.3 |
|  |  | [c.834_834＋1 del GG ins TT (splice)] | exon6-intron6 |  |  |  |  |  |  |  |  |  |
| P33 | HOGA1 | [c.834G>A (splice)] | exon6 | 2.5 | male | 96.84 | 104.89 | 309.14 | 0.32 | 174.99 | Ureteroscopy | 5.3 |
|  |  | [c.834_834＋1 del GG ins TT (splice)] | exon6-intron6 |  |  |  |  |  |  |  |  |  |
| P34 | HOGA1 | [c.554C>T (p.T185M)] | exon4 | 1.5 | female | 130.96 | 152.67 | 288.57 | 0.68 | 163.9 | None | 3.3 |
|  |  | [c.554C>T (p.T185M)] | exon4 |  |  |  |  |  |  |  |  |  |
| P35 | HOGA1 | [c.811C>T (p.R271C)] | exon6 | 5 | female | 128.87 | 110.47 | 54.76 | 0.36 | 105.13 | PCNL | 5.2 |
|  |  | [c.812G>A (p.R271H)] | exon6 |  |  |  |  |  |  |  |  |  |
| P36 | HOGA1 | [c.834G>A (splice)] | exon6 | 1.5 | male | 130.37 | 171.66 | 628.43 | 0.14 | 297.93 | PCNL | 3.8 |
|  |  | [c.834_834＋1 del GG ins TT (splice)] | exon6-intron6 |  |  |  |  |  |  |  |  |  |
| P37 | HOGA1 | [c.117delC (p. P41LfX2)] | exon1 | 0.7 | male | 62.58 | 59.86 | 906.77 | 0.13 | 160.93 | RIRS | 4.3 |
|  |  | [c.834G>A (splice)] | exon6 |  |  |  |  |  |  |  |  |  |
| P38 | HOGA1 | [c.554C>T (p.T185M)] | exon2 | 2 | male | 97.24 | 113.8 | 504.35 | 0.14 | 138.29 | Ureteroscopy | 6.6 |
|  |  | [c.769T>G (p.C257G)] | exon6 |  |  |  |  |  |  |  |  |  |
| P39 | HOGA1 | [c.346C>T (p.Q116*)] | exon3 | 0.8 | male | 60.84 | 53.89 | 333.7 | 0.32 | 252.97 | Ureteroscopy, RIRS | 4.8 |
|  |  | [c.834G>A (splice)] | exon6 |  |  |  |  |  |  |  |  |  |
| P40 | HOGA1 | [c.834G>A (splice)] | exon6 | 2 | male | 167.02 | 173.47 | 1052.55 | 0.21 | 171.86 | RIRS | 3.9 |
|  |  | [c.834G>A (splice)] | exon6 |  |  |  |  |  |  |  |  |  |
| P41 | HOGA1 | [c.834G>A (splice)] | exon6 | 0.4 | female | 97.67 | 137.82 | 1205.59 | 0.25 | 518.25 | RIRS | 3.9 |
|  |  | [c.834_834＋1 del GG ins TT (splice)] | exon6-intron6 |  |  |  |  |  |  |  |  |  |
| P42 | HOGA1 | [c.769T>G (p.C257G)] | exon6 | 0.4 | male | ND | ND | 18.57 | 2.43 | 89.76 | RIRS | 0.5 |
|  |  | [c.769T>G (p.C257G)] | exon6 |  |  |  |  |  |  |  |  |  |
| ND，not detected; UL, urolithiasis; UTI, urinary tract infection; RIRS, Retrograde intrarenal surgery; PCNL, Percutaneous nephrolithotomy | | | | | | | | | | | | |

| \| Supplementary Table S2. Allele Frequency of PH patients' mutations in this cohort and gnomAD database \| \| \| \| \| \| \| \| \| \| \| \| \| \| \| \| \| \| \| \| \| \| --- \| --- \| --- \| --- \| --- \| --- \| --- \| --- \| --- \| --- \| --- \| --- \| --- \| --- \| --- \| --- \| --- \| --- \| --- \| --- \| --- \| \| Gene \| Genotype \| Position \| Major/ minor haplotype^a^ \| Major/ minor haplotype^b^ \| This cohort \| SAS AC \| SAS AN \| SAS  AF \| EAS AC \| EAS AN \| EAS  AF \| NFE AC \| NFE  AN \| NFE  AF \| FIN AC \| FIN AN \| FIN  AF \| AFR AC \| AFR AN \| AFR  AF \| \| AGXT \| [c.33dup (p. K12QfX156)] \| exon 1 \| major \| major \| 22.22% \| 1 \| 4750 \| 0.0002105 \| 1 \| 5110 \| 0.0001957 \| 10 \| 67724 \| 0.0001477 \| 1 \| 10504 \| 0.0000952 \| 4 \| 40986 \| 0.00009759 \| \| AGXT \| [c.32C>G (p.P11R)] \| exon 1 \| major \| major \| 5.56% \| 2 \| 30536 \| 0.0000655 \| 28 \| 19896 \| 0.001407 \| 4 \| 125328 \| 0.0000399 \| 0 \| 24902 \| 0 \| 1 \| 24668 \| 0.00004054 \| \| AGXT \| [c.815_816insGA (p. L272RfX38)] \| exon 8 \| major \| major \| 16.67% \| 0 \| 30616 \| 0 \| 3 \| 18392 \| 0.0001631 \| 0 \| 113664 \| 0 \| 0 \| 21648 \| 0 \| 0 \| 16240 \| 0 \| \| AGXT \| [c.614C>T (p.S205L)] \| exon 6 \| major \| major \| 5.56% \| 0 \| 30616 \| 0 \| 0 \| 18386 \| 0 \| 1 \| 113534 \| 0.000008808 \| 0 \| 21592 \| 0 \| 0 \| 16236 \| 0 \| \| AGXT \| [c.1161C>A (p.C387*)] \| exon 11 \| major \| major \| 2.78% \| - \| - \| - \| - \| - \| - \| - \| - \| - \| - \| - \| - \| - \| - \| - \| \| AGXT \| [c.484G>A (p.V162M)] \| exon 4 \| major \| major \| 5.56% \| - \| - \| - \| - \| - \| - \| - \| - \| - \| - \| - \| - \| - \| - \| - \| \| AGXT \| [c.2T>C (p.M1T)] \| exon 1 \| major \| major \| 2.78% \| 0 \| 29932 \| 0 \| 9 \| 18044 \| 0.0004988 \| 1 \| 106978 \| 0.000009348 \| 0 \| 20736 \| 0 \| 1 \| 15162 \| 0.00006595 \| \| AGXT \| [c.605T>A (p.I202N)] \| exon 6 \| major \| major \| 2.78% \| - \| - \| - \| - \| - \| - \| - \| - \| - \| - \| - \| - \| - \| - \| - \| \| AGXT \| [c.466G>A (p.G156R)] \| exon 4 \| major \| major \| 2.78% \| 0 \| 27496 \| 0 \| 0 \| 17054 \| 0 \| 5 \| 101706 \| 0.00004916 \| 0 \| 18964 \| 0 \| 1 \| 14004 \| 0.00007141 \| \| AGXT \| [c.909del (p. A304HfX8)] \| exon 9 \| major \| major \| 2.78% \| - \| - \| - \| - \| - \| - \| - \| - \| - \| - \| - \| - \| - \| - \| - \| \| AGXT \| [c.28_c.29delCCinsA (p. P10TfX36)] \| exon 1 \| major \| minor \| 2.78% \| 1 \| 30486 \| 0.0000328 \| 3 \| 18342 \| 0.0001636 \| 4 \| 109932 \| 0.00003639 \| 0 \| 21466 \| 0 \| 0 \| 15894 \| 0 \| \| AGXT \| [c.638C>T (p.A213V)] \| exon 6 \| major \| major \| 2.78% \| 0 \| 30616 \| 0 \| 5 \| 18382 \| 0.000272 \| 0 \| 113546 \| 0 \| 0 \| 21596 \| 0 \| 0 \| 16246 \| 0 \| \| AGXT \| [c.481G>A (p.G161S)] \| exon 4 \| major \| major \| 2.78% \| 0 \| 27504 \| 0 \| 0 \| 17054 \| 0 \| 4 \| 101236 \| 0.00003951 \| 0 \| 19012 \| 0 \| 0 \| 14036 \| 0 \| \| AGXT \| [c.121G>A (p.G41R)] \| exon 1 \| major \| major \| 5.56% \| 1 \| 30600 \| 0.00003268 \| 0 \| 18350 \| 0 \| 2 \| 110444 \| 0.00001811 \| 0 \| 21560 \| 0 \| 0 \| 15764 \| 0 \| \| AGXT \| [c.145A>C (p.M49L)] \| exon 1 \| major \| major \| 2.78% \| 2 \| 30334 \| 0.00006593 \| 122 \| 18910 \| 0.006452 \| 8 \| 125636 \| 0.00006368 \| 0 \| 25008 \| 0 \| 0 \| 23138 \| 0 \| \| AGXT \| [c.679_680+2del (splice)] \| exon6-intron6 \| major \| major \| 5.56% \| 0 \| 30616 \| 0 \| 3 \| 18380 \| 0.0001632 \| 0 \| 113302 \| 0 \| 0 \| 21584 \| 0 \| 0 \| 16226 \| 0 \| \| AGXT \| [c.824G>A (p.S275N)] \| exon 8 \| major \| major \| 5.56% \| - \| - \| - \| - \| - \| - \| - \| - \| - \| - \| - \| - \| - \| - \| - \| \| GRHPR \| [c.181G>A (p.D61N)] \| exon 2 \| - \| - \| 7.14% \| 1 \| 30604 \| 0.00003268 \| 4 \| 18360 \| 0.0003268 \| 2 \| 111088 \| 0.000018 \| 0 \| 20006 \| 0 \| 0 \| 16166 \| 0 \| \| GRHPR \| [c.83+1G>C (splice)] \| intron 1 \| - \| - \| 7.14% \| - \| - \| - \| - \| - \| - \| - \| - \| - \| - \| - \| - \| - \| - \| - \| \| GRHPR \| [c.864_865del (p.C288CfX23)] \| exon 8 \| - \| - \| 71.43% \| 1 \| 30614 \| 0.00003266 \| 10 \| 19952 \| 0.0005012 \| 0 \| 129148 \| 0 \| 0 \| 25120 \| 0 \| 0 \| 24970 \| 0 \| \| GRHPR \| [c.419C>T (p.S140L)] \| exon 5 \| - \| - \| 14.29% \| 0 \| 30616 \| 0 \| 2 \| 18388 \| 0.0001088 \| 0 \| 113598 \| 0 \| 0 \| 21192 \| 0 \| 0 \| 16220 \| 0 \| \| HOGA1 \| [c.834_834＋1 del GG ins TT (splice)] \| exon6-intron6 \| - \| - \| 17.65% \| 3 \| 29716 \| 0.000101 \| 13 \| 17924 \| 0.0007253 \| 0 \| 108418 \| 0 \| 0 \| 21092 \| 0 \| 0 \| 14924 \| 0 \| \| HOGA1 \| [c.811C>T (p.R271C)] \| exon 6 \| - \| - \| 5.88% \| 15 \| 30200 \| 0.0004967 \| 2 \| 19710 \| 0.0001015 \| 5 \| 126458 \| 0.00003954 \| 1 \| 24830 \| 0.00004027 \| 0 \| 24392 \| 0 \| \| HOGA1 \| [c.834G>A (splice)] \| exon 6 \| - \| - \| 35.29% \| 2 \| 29730 \| 0.00006727 \| 17 \| 19484 \| 0.0008725 \| 6 \| 124018 \| 0.00004838 \| 0 \| 24564 \| 0 \| 0 \| 23704 \| 0 \| \| [c.952C>T (p.R318C)] \| exon 7 \| - \| - \| 2.94% \| 1 \| 30612 \| 0.00003267 \| 0 \| 19944 \| 0 \| 5 \| 128920 \| 0.00003878 \| 0 \| 24914 \| 0 \| 2 \| 24934 \| 0.00008021 \| \| HOGA1 \| [c.769T>G (p.C257G)] \| exon 6 \| - \| - \| 17.65% \| 0 \| 30486 \| 0 \| 18 \| 19878 \| 0.0009055 \| 0 \| 128074 \| 0 \| 0 \| 25004 \| 0 \| 0 \| 24770 \| 0 \| \| [c.812G>A (p.R271H)] \| exon 6 \| - \| - \| 5.88% \| 2 \| 30120 \| 0.0000664 \| 2 \| 18146 \| 0.0001102 \| 0 \| 110796 \| 0 \| 0 \| 21308 \| 0 \| 1 \| 15614 \| 0.00006405 \| \| HOGA1 \| [c.554C>T (p.T185M)] \| exon 4 \| - \| - \| 8.82% \| 5 \| 30616 \| 0.0001633 \| 101 \| 19954 \| 0.005062 \| 35 \| 129192 \| 0.0002709 \| 0 \| 25120 \| 0 \| 0 \| 24964 \| 0 \| \| HOGA1 \| [c.117delC (p. P41LfX2)] \| exon 1 \| - \| - \| 2.94% \| - \| - \| - \| - \| - \| - \| - \| - \| - \| - \| - \| - \| - \| - \| - \| \| HOGA1 \| [c.346C>T (p.Q116*)] \| exon 3 \| - \| - \| 2.94% \| 0 \| 30614 \| 0 \| 1 \| 18374 \| 0.00005442 \|  \| 113210 \| 0 \| 0 \| 20836 \| 0 \| 0 \| 16172 \| 0 \| \| AC: Allele Count; AF Allele Frequency; AN Allele Number; South Asian: SAS; East Asian: EAS; NFE: Non-Finnish European; FIN: Finnish European; AFR: African/African American a Minor allele defined as p. P11L.  b Minor allele defined as p. I340M. \| \| \| \| \| \| \| \| \| \| \| \| \| \| \| \| \| \| \| \| \| |
| --- | --- | --- | --- | --- | --- | --- | --- | --- | --- | --- | --- | --- | --- | --- | --- | --- | --- | --- | --- | --- | --- | --- | --- | --- | --- | --- | --- | --- | --- | --- | --- | --- | --- | --- | --- | --- | --- | --- | --- | --- | --- | --- | --- | --- | --- | --- | --- | --- | --- | --- | --- | --- | --- | --- | --- | --- | --- | --- | --- | --- | --- | --- | --- | --- | --- | --- | --- | --- | --- | --- | --- | --- | --- | --- | --- | --- | --- | --- | --- | --- | --- | --- | --- | --- | --- | --- | --- | --- | --- | --- | --- | --- | --- | --- | --- | --- | --- | --- | --- | --- | --- | --- | --- | --- | --- | --- | --- | --- | --- | --- | --- | --- | --- | --- | --- | --- | --- | --- | --- | --- | --- | --- | --- | --- | --- | --- | --- | --- | --- | --- | --- | --- | --- | --- | --- | --- | --- | --- | --- | --- | --- | --- | --- | --- | --- | --- | --- | --- | --- | --- | --- | --- | --- | --- | --- | --- | --- | --- | --- | --- | --- | --- | --- | --- | --- | --- | --- | --- | --- | --- | --- | --- | --- | --- | --- | --- | --- | --- | --- | --- | --- | --- | --- | --- | --- | --- | --- | --- | --- | --- | --- | --- | --- | --- | --- | --- | --- | --- | --- | --- | --- | --- | --- | --- | --- | --- | --- | --- | --- | --- | --- | --- | --- | --- | --- | --- | --- | --- | --- | --- | --- | --- | --- | --- | --- | --- | --- | --- | --- | --- | --- | --- | --- | --- | --- | --- | --- | --- | --- | --- | --- | --- | --- | --- | --- | --- | --- | --- | --- | --- | --- | --- | --- | --- | --- | --- | --- | --- | --- | --- | --- | --- | --- | --- | --- | --- | --- | --- | --- | --- | --- | --- | --- | --- | --- | --- | --- | --- | --- | --- | --- | --- | --- | --- | --- | --- | --- | --- | --- | --- | --- | --- | --- | --- | --- | --- | --- | --- | --- | --- | --- | --- | --- | --- | --- | --- | --- | --- | --- | --- | --- | --- | --- | --- | --- | --- | --- | --- | --- | --- | --- | --- | --- | --- | --- | --- | --- | --- | --- | --- | --- | --- | --- | --- | --- | --- | --- | --- | --- | --- | --- | --- | --- | --- | --- | --- | --- | --- | --- | --- | --- | --- | --- | --- | --- | --- | --- | --- | --- | --- | --- | --- | --- | --- | --- | --- | --- | --- | --- | --- | --- | --- | --- | --- | --- | --- | --- | --- | --- | --- | --- | --- | --- | --- | --- | --- | --- | --- | --- | --- | --- | --- | --- | --- | --- | --- | --- | --- | --- | --- | --- | --- | --- | --- | --- | --- | --- | --- | --- | --- | --- | --- | --- | --- | --- | --- | --- | --- | --- | --- | --- | --- | --- | --- | --- | --- | --- | --- | --- | --- | --- | --- | --- | --- | --- | --- | --- | --- | --- | --- | --- | --- | --- | --- | --- | --- | --- | --- | --- | --- | --- | --- | --- | --- | --- | --- | --- | --- | --- | --- | --- | --- | --- | --- | --- | --- | --- | --- | --- | --- | --- | --- | --- | --- | --- | --- | --- | --- | --- | --- | --- | --- | --- | --- | --- | --- | --- | --- | --- | --- | --- | --- | --- | --- | --- | --- | --- | --- | --- | --- | --- | --- | --- | --- | --- | --- | --- | --- | --- | --- | --- | --- | --- | --- | --- | --- | --- | --- | --- | --- | --- | --- | --- | --- | --- | --- | --- | --- | --- | --- | --- | --- | --- | --- | --- | --- | --- | --- | --- | --- | --- | --- | --- | --- | --- | --- | --- | --- | --- | --- | --- | --- | --- | --- | --- | --- | --- | --- | --- | --- | --- | --- | --- | --- | --- | --- | --- | --- | --- | --- | --- | --- | --- | --- | --- | --- | --- | --- | --- | --- | --- | --- | --- | --- | --- | --- | --- | --- | --- | --- | --- | --- | --- | --- | --- | --- | --- | --- | --- | --- | --- | --- | --- | --- | --- | --- | --- | --- | --- | --- | --- | --- | --- | --- | --- | --- | --- | --- | --- | --- | --- | --- | --- | --- | --- | --- | --- | --- | --- | --- | --- | --- | --- | --- | --- | --- | --- | --- | --- | --- | --- | --- | --- | --- | --- | --- | --- | --- | --- | --- | --- | --- | --- | --- | --- | --- | --- | --- | --- | --- | --- | --- | --- | --- | --- | --- | --- | --- | --- | --- | --- | --- | --- | --- | --- | --- | --- | --- | --- | --- | --- | --- | --- | --- | --- | --- | --- | --- | --- | --- | --- |

| Supplementary Table S3. Phenotypes of PH1 patients with splicing/nonsense/frameshift mutations and missense mutations | | | |
| --- | --- | --- | --- |
| Variables | splicing/nonsense/frameshift mutations (n=12) | missense mutation (n=10) | P |
| Age of onset (years) | 5.30±3.19 (n=12) | 4.69±3.74 (n=10) | 0.684 |
| Nephrocalcinosis (%) | 91.67% (n=11) | 40.00% (n=4) | 0.02* |
| eGFR (ml/min/1.73 m^2^) | 79.76±25.67 (n=11) | 124.18±32.56 (n=9) | 0.003* |
| Urine oxalate/creatinine (μg/mg)  (nL<253.88) | 712.44±586.38 (n=8) | 354.60±309.10 (n=9) | 0.130 |
| Urine calcium/creatinine (mmol/mmol)  (nL<0.57) | 0.23±0.31  (n=7) | 0.37±0.39 (n=9) | 0.299 |
| Urine citrate/creatinine (μg/mg)  (nL>=45.62) | 180.54±165.52 (n=7) | 123.56±86.97 (n=7) | 0.436 |
| Abbreviations: nL, normal limit; eGFR: estimated glomerular filtration rate | | | |

|  |  |  |  |
| --- | --- | --- | --- |
| Supplementary Table S4. Phenotypes of PH2 patients with splicing/nonsense/frameshift mutations and missense mutations | | | |
| Variables | splicing/nonsense/frameshift mutations (n=7) | missense mutation (n=3) | P |
| Age of onset (years) | 7.43±3.74 (n=7) | 9.67±3.06 (n=3) | 0.391 |
| Nephrocalcinosis (%) | 42.86% (n=3) | 66.67% (n=2) | 1.000 |
| eGFR (ml/min/1.73 m^2^) | 109.96±27.61 (n=7) | 97.00±38.51 (n=3) | 0.557 |
| Urine oxalate/creatinine (μg/mg)  (nL<253.88) | 186.84±165.82 (n=6) | 176.35±164.66 (n=3) | 0.931 |
| Urine calcium/creatinine (mmol/mmol)  (nL<0.57) | 0.20±0.15 (n=5) | 0.06±0.06 (n=2) | 0.272 |
| Urine citrate/creatinine (μg/mg)  (nL>=45.62) | 94.96±73.50 (n=6) | 61.69±60.87 (n=3) | 0.524 |
| Abbreviations: nL, normal limit; eGFR: estimated glomerular filtration rate | | | |

|  |  |  |  |
| --- | --- | --- | --- |
| Supplementary Table S5. Phenotypes of PH3 patients with splicing/nonsense/frameshift mutations and missense mutations | | | |
| Variables | splicing/nonsense/frameshift mutations (n=11) | missense mutation (n=8) | P |
| Age of onset (years) | 1.52±1.63 (n=11) | 1.53±1.64 (n=8) | 0.84 |
| Nephrocalcinosis (%) | 27.27% (n=3) | 25.00% (n=2) | 1.000 |
| eGFR (ml/min/1.73 m^2^) | 98.77±31.18 (n=11) | 96.11±27.55 (n=7) | 0.856 |
| Urine oxalate/creatinine (μg/mg)  (nL<253.88) | 503.47±394.36 (n=11) | 360.27±473.33 (n=8) | 0.238 |
| Urine calcium/creatinine (mmol/mmol)  (nL<0.57) | 0.32(0.13, 1.30) (n=11) | 0.74±0.79 (n=8) | 0.657 |
| Urine citrate/creatinine (μg/mg)  (nL>=45.62) | 231.59±133.40 (n=11) | 183.57±194.08  (n=8) | 0.109 |
| Abbreviations: nL, normal limit; eGFR: estimated glomerular filtration rate | | | |
